# Evaluation of Sanitizing Methods for Reducing Microbial Contamination on Fresh Strawberry, Cherry Tomato, and Red Bayberry

**DOI:** 10.3389/fmicb.2017.02397

**Published:** 2017-12-05

**Authors:** Wei Wei, Xu Wang, Zhongwen Xie, Wen Wang, Junfeng Xu, Yuanjing Liu, Haiyan Gao, Yu Zhou

**Affiliations:** ^1^State Key Laboratory of Tea Plant Biology and Utilization, School of Tea and Food Science and Technology, Anhui Agricultural University, Hefei, China; ^2^State Key Laboratory Breeding Base for Zhejiang Sustainable Pest and Disease Control, Institute of Quality and Standard for Agro-products, Zhejiang Academy of Agricultural Sciences, Hangzhou, China

**Keywords:** fresh produce, sodium hypochlorite, organic acid, acidified sodium chlorite, *Escherichia coli*, *Salmonella* Typhimurium

## Abstract

Strawberries, cherry tomatoes, and red bayberries, which are the most popular types of fresh produce in China, are vulnerable to microbial contamination. In this study, different sanitizing methods [treatment with 2% organic acids, 0.02% sodium hypochlorite (SH), 0.1% sodium chlorite (SC), and 0.1% acidified sodium chlorite (ASC)] were applied to fresh strawberry, cherry tomato, and red bayberry, and their abilities to reduce aerobic bacteria, *Escherichia coli* O157:H7, mold, yeast, and *Salmonella* Typhimurium were evaluated. The commercially used SH method reduced the background microbiota on strawberry, cherry tomato, and red bayberry by 0.20–2.07 log cfu/g. The ASC method reduced background microbiota (except for mold) on strawberry and cherry tomato by more than 3.0 log cfu/g. ASC was the only sanitizer that significantly reduced mold on red bayberry, and lactic acid was the only organic acid sanitizer that effectively reduced yeast on red bayberry. The ASC method had the best sterilizing effect on the three fresh fruits and also required the shortest sanitizing time and low chlorite content. The application of ASC method significantly reduced the microbiota on retail grocery samples, and the effect was similar to that achieved by sanitizing methods comparison.

## Introduction

In terms of cultivated acreage and production, China is one of the world’s largest producers of strawberry, cherry tomato, and red bayberry. However, because of their high contents of water and carbohydrates along with thin skins, fresh produce is vulnerable to physical damage and microbial contamination during harvest and transportation (Beuchat, 1996). The outbreaks of *Escherichia coli* O157 infections in the United States and Germany in recent years were attributed to the pathogenic contamination of strawberries, spinach, and cucumbers, and these kinds of incident have increased year by year (Laidler et al., 2013). *Salmonella* spp. has caused considerable concern in the food industry as it has been associated with large-scale outbreaks of food-borne illness (Tauxe et al., 2010). The primary organisms that cause post-harvest spoilage in fresh produce are gray mold (*Botrytis cinerea* Pers. ex. Fr.) and rhizopus rot (*Rhizopus stolonifer* Ehrenb. Fr. Vuill) (Vardar et al., 2012). Because of the low pH values of many fruits, especially red bayberry, the predominant microbiota consists of molds and yeasts (Oms-Oliu et al., 2010). Pathogens such as *Salmonella* spp. and *E. coli* often contaminate ground-growing fruits with relatively high pH (e.g., strawberry and cherry tomato) (Oms-Oliu et al., 2010). As red bayberry grows on a tree, it is not contaminated by *Salmonella.*

**FIGURE 1:**
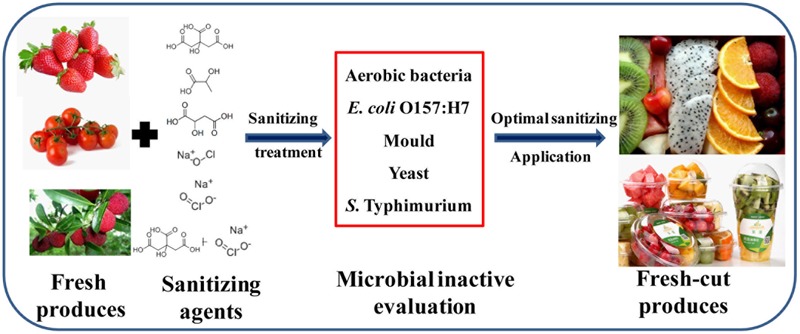
Flowchart showing the processing steps for fresh produce.

To reduce microbial contamination, washing is a key sanitization step in the fresh-cut produce production chain (from farmland to dining table, **Figure 1**) (Meireles et al., 2016). Many sanitizers have been evaluated for their ability to inactivate pathogens on fresh-cut fruits and vegetables (Oms-Oliu et al., 2010; Abadiasa et al., 2011; Keskinen and Annous, 2011; Luo and Oh, 2016; Oladunjoye et al., 2016). SH at concentrations of 50–200 mg/L is commonly used as a commercial sanitizer to remove microbiota from fresh fruits, vegetables, and fresh-cut produce (Beuchat et al., 1998). As a United States FDA-accepted food sanitizer, SC is frequently selected as the sanitizer for washing fresh produce (Meireles et al., 2016). Several studies indicated that washing with 0.2% SC significantly reduced *Listeria monocytogenes* and *E. coli* O157:H7 on shredded lettuce and cabbage (Zhang and Farber, 1996; Takeuchi and Frank, 2001; Van Haute et al., 2017). However, the use of chlorine in food products is limited because of its toxic by-products, including trihalomethane and chlorite residues (Richardson et al., 1998; Gil et al., 2016). Organic acid sanitizers (e.g., CA, LA, and MA), which are considered “GRAS” by the FDA, have been evaluated in various studies and approved for use in foods (Chen et al., 2016). Organic acids are unambiguously able to inactivate food-borne pathogens (Akbas and Olmez, 2007), and 2% is generally considered as the appropriate concentration for reducing *E. coli* O157:H7, *Salmonella* Typhimurium, and *L. monocytogenes* (Sagong et al., 2011; Neal et al., 2012; Wang et al., 2013; Ramos-Villarroel et al., 2015; Salinas-Roca et al., 2016). The disadvantages of organic acids include high cost, odor, and corrosiveness. Thus, it is desirable to reduce the organic acid concentration while simultaneously increasing the antimicrobial effect. ASC refers to an aqueous solution of SC containing any GRAS acid. ASC containing CA (pH 2.5–3.2) was reported as a highly effective antimicrobial agent for fresh produce (Warf, 2001). The combination of SC and CA produces active ClO_2_, which is more soluble in water than SH and has an oxidizing capacity two times greater than that of hypochlorite (ClO^-^); the ASC is more effective at reducing natural microbiota and food-borne pathogens on fresh-cut cilantro and Chinese cabbage (Inatsu et al., 2005; Allende et al., 2009; Prado-Silva et al., 2015). FDA approved ASC containing 0.5–1.2 g/L SC as a candidate for spraying or dipping fresh produce during processing (Code of Federal Regulations, 2000).

The EFSA Opinions on *Salmonella* spp. and Norovirus disinfection discuss the sanitization of tomatoes using ClO_2_ [EFSA Panel on Biological Hazards (BIOHAZ), 2014]. The *Salmonella* spp. contamination of fresh tomatoes was found to be effectively eliminated by 3 mg/L ClO_2_, and the sanitizing ability was closely related to water turbidity and temperature [López-Velasco et al., 2012; EFSA Panel on Biological Hazards (BIOHAZ), 2014]. However, other effective methods for preventing the microbial contamination of strawberry, cherry tomato, and red bayberry have rarely been investigated. In this study, several methods that are effective for sanitizing other types of fresh produce were applied to the above three fresh fruits, and the results were compared. Specifically, the optimal concentrations and washing times for sanitizing agents (organic acids, SH, SC, and ASC) determined in previous studies were used to sanitize fresh strawberry, cherry tomato, and red bayberry, and the best method for reducing microbial populations (i.e., aerobic bacteria, *E. coli*, yeast, mold, and *S.* Typhimurium) on each produce type was determined.

## Materials and Methods

### Sample Collection

Strawberry (*Fragaria* × *ananassa* Duch) and cherry tomato (*Lycopersicon esculentum* var. *cerasiforme*) produced by the Shenboleyuan farm in Hangzhou (Zhejiang, China) and red bayberry (*Myrica rubra*) produced by the Shenxianju garden in Xianju (Zhejiang, China) were selected for evaluation. A total of 3 kg of each type of produce was collected when the fruit maturity was approximately 80%, as indicated by a red surface color. For the microbial safety survey, fresh strawberry, cherry tomato, and red bayberry free of external defects were randomly purchased from local retail grocers, supermarkets, and fruit shops in Hangzhou (Zhejiang, China) on their day of their arrival from the producers. A total of 50 samples (1 kg each) of each fruit were collected and prepared for the comparison of microbiota before and after sanitizing treatment. The collected fruits were evaluated for microbial content in terms of total aerobic bacteria, *E. coli*, yeast, mold, and *S.* Typhimurium. The collected samples were transported immediately to the laboratory under refrigerated conditions. The samples were stored at 4°C in the laboratory, and the experimental treatments were completed within 24 h.

### Preparation and Inoculation of Bacterial Strains

The *E. coli* O157:H7 (ATCC 43895) and *S.* Typhimurium (ATCC 14028) bacterial strains used in this study were preserved cultures originally obtained from the American Type Culture Collection (ATCC). The *E. coli* O157:H7 (E8) and *S.* Typhimurium (S9) strains were isolated from chickens in our laboratory. The strains of *E. coli* O157:H7 and *S.* Typhimurium were kept at -80°C in Luria-Bertani broth (LB, Difco) containing 20% (v/v) glycerol for long-term preservation and maintained on LB slants at 4°C for temporary use. For experimental preparation, a loop inoculum was transferred to LB broth and incubated for 24 h at 37°C for each food-borne microbial strain. Consecutive loop transfers of 24 h LB cultures were made, and cocktails of *E. coli* O157:H7 (ATCC 43895 and E8) and *S.* Typhimurium (ATCC 14028 and S9) were prepared from equal volumes of each strain suspension. The cocktail of each pathogen was used as the inoculum for the evaluation of each sanitizing method. The inoculum concentrations of *E. coli* O157:H7 and *S.* Typhimurium were standardized at 10^7^ cfu/mL by diluting with sterile 0.1% peptone water. The inoculum suspension of *E. coli* O157:H7 was applied to the strawberry, cherry tomato, and red bayberry samples, and the inoculum suspension of *S.* Typhimurium was applied to the strawberry and cherry tomato samples. Mesh bags containing the fresh fruits were submerged in the inoculum suspensions and gently agitated for 30 min. After inoculation, the produce samples were air-dried in a biosafety chamber (AIRTECH, Suzhou, China) for 30 min to facilitate bacterial adhesion before exposure to various disinfection treatments (Wang et al., 2013). All work involving biohazardous agents was performed under Biosefety Level 2 conditions.

### Sanitizing Treatment

The chemicals used to prepare the sanitizers were obtained from Sigma–Aldrich (Shanghai, China), and fresh sanitizing solutions were prepared with deionized water. The optimal sanitizing times and concentrations of the previously described sanitizers were applied to the three types of fresh produce in this study (Allende et al., 2009; Abadiasa et al., 2011; Sagong et al., 2011; Neal et al., 2012; Wang et al., 2013; Chen et al., 2016). The selected sanitizing solutions of 0.02% SH (200 mg/L free chlorine, pH 6.5), 0.1% SC (1.0 g/L, pH 9.4), 2% CA (20 g/L, pH 2.1), 2% LA (20 mL/L, pH 2.1), 2% MA (20 g/L, pH 3.3), and 0.1% ASC (containing 1.0 g/L SC, pH 2.3) were prepared according to previously reported methods (Allende et al., 2009; Abadiasa et al., 2011; Sagong et al., 2011; Neal et al., 2012; Wang et al., 2013; Chen et al., 2016). The sanitizing solutions were prepared immediately before use, and TW (pH 6.8) was used as the control. After the inoculation step (i.e., *E. coli* O157:H7 and *S.* Typhimurium), the sample contained in a mesh bag was dipped into the sanitizing solution for the amount of time specified in the relevant previous study (**Table 1**). Subsequently, the sample was transferred to a new sterile bag and rinsed with 100 mL sterile water. The mesh bag was gently massaged by hand for 1 min to remove chemical residues that might interfere with subsequent microbial analysis. To evaluate the sanitizing treatments against natural microbiota (i.e., aerobic bacteria, mold, and yeast), fresh produce samples were randomly selected, and the sanitizing treatments were performed without the artificial inoculation step. The microbiota populations were determined before and after the sanitizing treatments, and the *D*-values were calculated.

**Table 1:** Sanitizing solutions and treatment conditions in this study.

Sanitizing agent	Concentration	pH	Sanitizing time (min)
TW	–	6.8	2
TW	–	6.8	10
SH	200 mg/L (0.02%)	6.5	2
CA	20 g/L (2%)	2.1	10
LA	20 mL/L (2%)	2.1	10
MA	20 g/L (2%)	3.3	10
SC	1.0 g/L (0.1%)	9.4	10
ASC	1.0 g/L (0.1%)	2.3	2

### Microbial Plate Counting

A 25-g portion of fresh produce was homogenized in 225 mL sterile physiological saline (8.5 g/L of NaCl) for 1 min using a blender (Stomacher 400, Seward, London, United Kingdom). Serial, 10-fold dilutions of each homogenate were prepared with sterile 0.1% peptone water, and 0.1 mL volumes of undiluted or suitable dilutions of the homogenate were spread on triplicate plates of the corresponding medium. Specifically, aerobic bacteria were incubated on plate count agar (Hope-Bio, China) at 30°C for 48 h; yeast and mold were incubated on potato dextrose agar (Hope-Bio, China) at 28°C for 48–72 h (Murray et al., 2007). *E. coli* O157:H7 and *S.* Typhimurium were determined by plating dilutions onto LB agar (Difco) or xylose lysine deoxycholate agar (Difco) at 37°C for 24 h. The culture purity of *E. coli* O157:H7 and *S.* Typhimurium was confirmed by Gram staining and biochemical tests (Smibert and Krieg, 1994). After incubation, colony counting was carried out using an automatic bacteria counter (Shineso Science and Technology Co., Ltd., China).

### The Optimal Sanitizing Method Application to Retail Grocers Samples

A total of 50 samples of each fruit type were subjected to the optimal sanitizing methods determined from the evaluations of the methods, and the effects of the optimal sanitizing methods on microbial contamination were evaluated. The strawberry and cherry tomato samples were evaluated for total aerobic bacteria, *E. coli*, yeast, mold, and *S.* Typhimurium, and the red bayberry samples were evaluated for total aerobic bacteria, *E. coli*, yeast, and mold.

### Statistical Analysis

All the experiments of this study were performed in triplicate (i.e., three independent tests) with each independent test containing three replica platings. The average plate count from each treatment was converted to log cfu/g. GraphPad Prism software was used for data analysis. The reduction in microbiota or pathogens was the *D*-value obtained for no treatment and for sanitizing treatment. Data are presented as means ± SD. Analyses were assessed by Student’s *t*-test, and *p*-values < 0.05 were considered statistically significant.

## Results and Discussion

### Washing with TW

**FIGURE 2:**
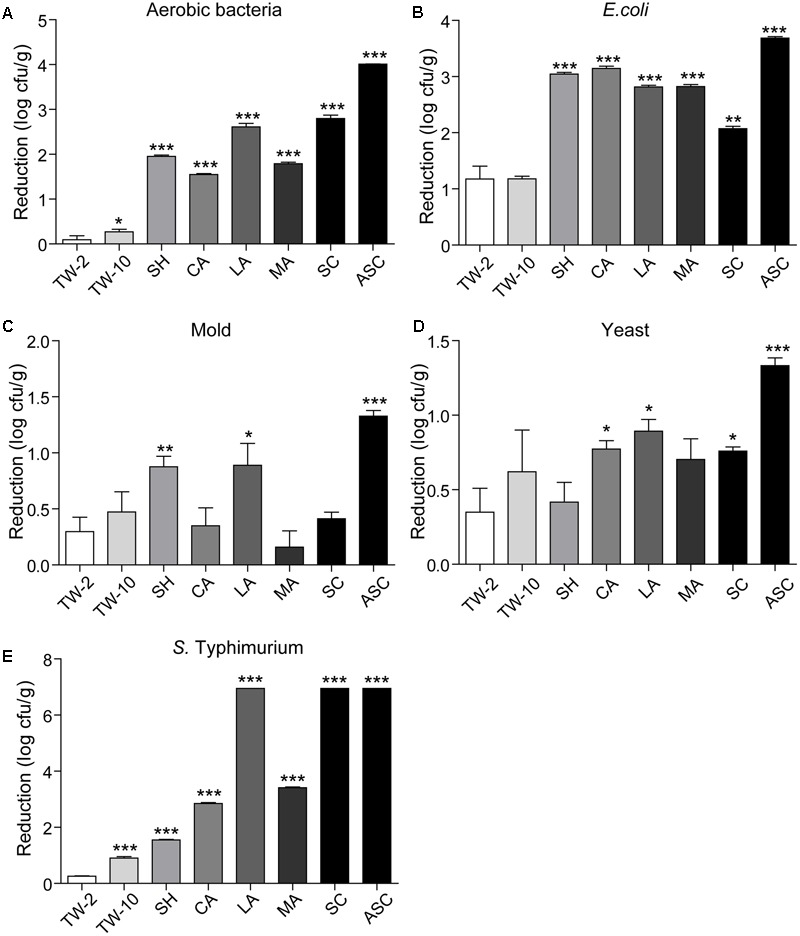
Reductions in aerobic bacteria **(A)**, *E. coli* **(B)**, mold **(C)**, yeast **(D)**, and *S.* Typhimurium **(E)** on strawberry achieved by different sanitizing methods. Data are expressed as mean ± SD (*n* = 3). ^∗^*P* < 0.05, ^∗∗^*P* < 0.01, and ^∗∗∗^*P* < 0.001 versus TW-2.

**FIGURE 3:**
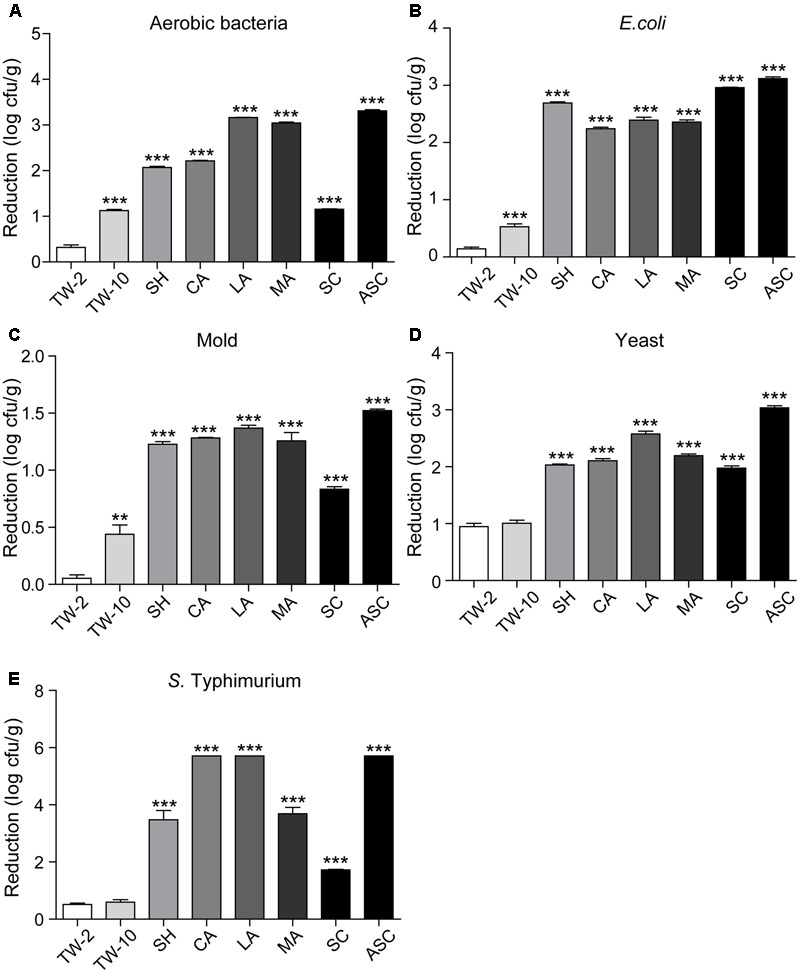
Reductions in aerobic bacteria **(A)**, *E. coli* **(B)**, mold **(C)**, yeast **(D)**, and *S.* Typhimurium **(E)** on cherry tomato achieved by different sanitizing methods. Data are expressed as mean ± SD (*n* = 3). ^∗∗^*P* < 0.01 and ^∗∗∗^*P* < 0.001 versus TW-2.

**FIGURE 4:**
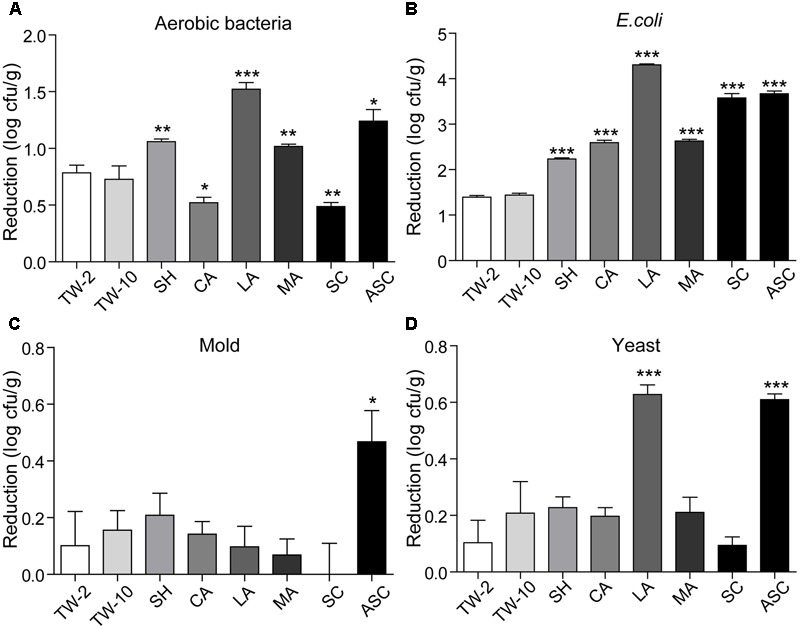
Reductions in aerobic bacteria **(A)**, *E. coli* **(B)**, mold **(C)**, and yeast **(D)** on red bayberry achieved by different sanitizing methods. Data are expressed as mean ± SD (*n* = 3). ^∗^*P* < 0.05, ^∗∗^*P* < 0.01, and ^∗∗∗^*P* < 0.001 versus TW-2.

Washing the surfaces of the fruits with TW is the method most frequently used by consumers to remove soil and some microorganisms; however, the efficacy of this method is limited (Neal et al., 2012). In this study, washing with TW for 10 min (TW-10) significantly reduced the amounts of aerobic bacteria and *S.* Typhimurium on strawberries compared to the control (washing with TW for 2 min; TW-2), but it did not significantly reduce *E. coli* O157:H7, mold, or yeast (**Figure 2**). In cherry tomato, TW-10 treatment significantly reduced aerobic bacteria, *E. coli* O157:H7, and mold on cherry tomato (**Figure 3**) but did not produce significant reductions in any microbiota or pathogen on red bayberry (**Figure 4**). The reductions in microbiota and pathogens resulting from TW-10 treatment were lower than 1.5 (0.15–1.44) log cfu/g, consistent with the results of Neal et al. (2012) (0.7 log cfu/g), Keskinen and Annous (2011) (0.77 log cfu/g), and others (Abadiasa et al., 2011; Wang et al., 2013). These results indicate that TW treatment is not sufficient for sanitizing strawberry, cherry tomato, and red bayberry. Generally, the microbial reductions achieved by washing with water were higher for strawberry and cherry tomato than for red bayberry; this may be attributed to the glossy surfaces of strawberries and cherry tomatoes (Velázquez et al., 2009).

### Treatment with Organic Acids

Organic acids (LA, CA, and MA) that are recognized as GRAS agents (Chen et al., 2016) were evaluated for their ability to sanitize fresh produce. For strawberry, treatment with LA solution significantly reduced all microbiota and pathogens; treatment with CA solution significantly reduced aerobic bacteria, *E. coli* O157:H7, yeast, and *S.* Typhimurium but not mold; and treatment with MA solution significantly reduced aerobic bacteria, *E. coli* O157:H7, and *S.* Typhimurium but not mold and yeast (**Figure 2**). For cherry tomato, the three organic acids produced significant reductions in the tested microbiota and pathogens (**Figure 3**). For red bayberry, the three organic acids produced significant reductions in *E. coli* O157:H7; although treatment with LA and MA significantly reduced aerobic bacteria, the aerobic bacteria reduction achieved by CA was significantly lower than the reduction observed for the control treatment (TW-2). LA was the only organic acid effective at reducing yeast, and no tested organic acid effectively reduced mold on red bayberry (**Figure 4**).

With the exceptions of mold on red bayberry and *E. coli* O157:H7 on strawberry, LA produced the largest reductions in microbiota and pathogens among the three organic acids. LA and CA resulted in the greatest reductions in *S.* Typhimurium (5.71 log cfu/g; *S.* Typhimurium was completely removed) on cherry tomato among the organic acid treatments. Moreover, LA produced the largest reductions in *E. coli* O157:H7 on red bayberry (4.31 log cfu/g) and *S.* Typhimurium on strawberry (6.95 log cfu/g) among all the sanitizing treatments in this study (**Table 2**). Previous studies reported that treatment with 2% LA reduced *E. coli* O157:H7 by 1.74 log cfu/g and *S.* Typhimurium by 1.73 log cfu/g on fresh organic lettuce (Sagong et al., 2011). Treatment with 2% LA at 55°C was reported to reduce *E. coli* O157:H7 and *Salmonella* spp. on fresh spinach leaves by 2.7 log cfu/g and 2.3 log cfu/g, respectively (Neal et al., 2012). Wang et al. (2013) found that treatment with 2% LA reduced *E. coli* O157:H7, *S.* Typhimurium, and *L. monocytogenes* by approximately 3.0 log cfu/g on minimally processed lotus sprouts, and the sanitizing efficiency of different treatments followed the approximate order of 2% LA > 1% LA > 0.5% LA > SH. In this study, the inoculated *E. coli* O157:H7 and *S.* Typhimurium were efficiently removed (2.39–6.95 log cfu/g) on the three fresh fruits by sanitizing with 2% LA, and the results were more efficient than those of on other previous investigations (Sagong et al., 2011; Neal et al., 2012; Wang et al., 2013).

**Table 2:** The largest reductions (log cfu/g) and corresponding sanitizing methods for different microbes/pathogens on fresh strawberry, cherry tomato, and red bayberry.

Sanitizer type	Fresh produce	Aerobic bacteria	*E. coli* O157:H7	Yeast	Mold	*S.* Typhimurium
Organic Acids	Strawberry	2.61 ± 0.14 /LA^b^	3.14 ± 0.07 /CA^b^	0.89 ± 0.14 /LA^b^	0.89 ± 0.34 /LA^b^	**6.95 ± 0.00 /LA^a^**
	Cherry tomato	3.16 ± 0.01 /LA^b^	2.39 ± 0.09 /LA^b^	2.58 ± 0.09 /LA^b^	1.37 ± 0.04 /LA^b^	5.71 ± 0.00 /CA or LA^b^
	Red bayberry	1.52 ± 0. 10 /LA^b^	**4.31 ± 0.03 /LA^a^**	0.63 ± 0.06 /LA^b^	0.14 ± 0.08 /CA^b^	ND
Chloric sanitizers	Strawberry	**4.01 ± 0.01 /ASC^a^**	3.68 ± 0.05 /ASC^c^	1.33 ± 0.09 /ASC^c^	1.33 ± 0.09 /ASC^c^	**6.95 ± 0.00 /SC or ASC^a^**
	Cherry tomato	3.31 ± 0.04 /ASC^c^	3.12 ± 0.05 /ASC^c^	**3.04 ± 0.06 /ASC^a^**	**1.52 ± 0.02 /ASC^a^**	5.71 ± 0.00 /ASC^c^
	Red bayberry	1.24 ± 0.30 /ASC^c^	3.67 ± 0.11 /ASC^c^	0.61 ± 0.03 /ASC^c^	0.47 ± 0.19 /ASC^c^	ND

*^a^The largest reduction among all sanitizing methods in this study; ^b^The largest reduction among organic acid sanitizing methods in this study; ^c^The largest reduction among chloric sanitizing methods in this study. LA, CA, or ASC under the reduction value indicates the corresponding sanitizing method; ND indicates not determined.*

### Treatment with SH, SC, and ASC

Compared to TW-2, sanitizing with SH resulted in significant reductions in aerobic bacteria, *E. coli* O157:H7, mold, and *S.* Typhimurium on strawberry (**Figure 2**). SH produced significant reductions in all microbiota and pathogens on cherry tomato (**Figure 3**) and significantly reduced aerobic bacteria and *E. coli* O157:H7 on red bayberry (**Figure 4**). However, yeast on strawberry along with yeast and mold on red bayberry were not significantly removed by sanitizing with SH (*p* > 0.05).

Sanitizing with SC resulted in significant reductions in aerobic bacteria, *E. coli* O157:H7, yeast, and *S.* Typhimurium on strawberry (**Figure 2**); significant reductions in all microbiota and pathogens on cherry tomato (**Figure 3**); and significant reductions in *E. coli* O157:H7 on red bayberry (**Figure 4**). However, the ability of SC to reduce aerobic bacteria was lower than that of the control treatment (TW-2) for red bayberry (**Figure 4**). Mold on strawberry along with yeast and mold on red bayberry were not significantly reduced by sanitizing with SC (*p* > 0.05). Comparatively, all microorganisms (i.e., microbiota and pathogens) on strawberry, cherry tomato, and red bayberry were significantly removed by treatment with 0.1% ASC. Thus, in this study, the efficiency of the different treatments decreased in the following approximate order: 0.1% ASC > 0.02% SH > 0.1% SC. The antimicrobial activity of ASC was much higher than those of SC and SH for the surface sterilization of strawberry and cherry tomato (**Figures 2, 3**).

Acidified sodium chlorite produced the largest reductions in microbiota and pathogens among the chloric sanitizing methods. ASC and SC generated the largest reductions (6.95 log cfu/g) in *S.* Typhimurium on strawberry among all tested sanitization methods. Moreover, ASC also produced the greatest reductions in aerobic bacteria (4.01 log cfu/g) on strawberry and yeast (3.04 log cfu/g) and mold (1.52 log cfu/g) on cherry tomato (**Table 2**). The inoculated *E. coli* O157:H7 and *S.* Typhimurium on strawberry and cherry tomato were completely inactivated by 0.1% ASC. Previous studies showed similar results as those obtained for red bayberry in this study. For example, 0.02% SH reduced microbiota and pathogens by 1.0–1.3 log cfu/g on fresh-cut cilantro, reduced pathogens by 1.0–1.5 log cfu/g on fresh spinach leaves, and reduced pathogens by approximately 0.5–1.0 log cfu/g on fresh-cut apple (Allende et al., 2009; Abadiasa et al., 2011; Neal et al., 2012). However, the reductions obtained on red bayberry in this study were clearly lower than those obtained for strawberry and cherry tomato in this study and the reductions reported in the past studies mentioned above. This discrepancy suggests that the nature of the fruit surface can strongly influence the sanitizing effect (Velázquez et al., 2009). For fresh-cut cilantro, treatment with 0.025 and 0.05% ASC reduced microbial populations by approximately 2 log cfu/g, while the reduction achieved by 0.1% ASC was more than 3 log cfu/g (Allende et al., 2009). Although 0.1% SC was as effective as 0.1% ASC in reducing aerobic bacteria and *E. coli* O157:H7, SC was not as effective as ASC in reducing yeast and mold populations on cut cilantro (Allende et al., 2009). In this study, the ability of ASC to reduce aerobic bacteria, *E. coli* O157:H7 and yeast on red bayberry was similar to that of LA; however, only ASC significantly reduced the mold population. In general, 0.1% ASC and 2% LA were the most effective treatments for the three types of produce in this study, and treatment with 0.1% ASC was optimal because of the shorter sanitizing time and lower SC content.

### The Application Results of ASC Sanitizing Method

**FIGURE 5:**
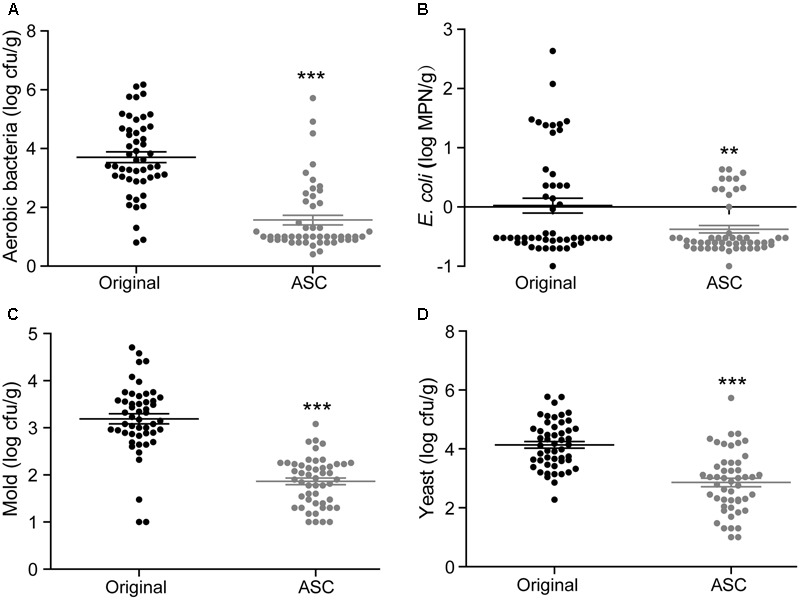
Effects of 0.1% ASC treatment on aerobic bacteria **(A)**, *E. coli* **(B)**, mold **(C)**, and yeast **(D)** for 50 randomly collected strawberry samples. Data are expressed as mean ± SD (*n* = 50). ^∗∗^*P* < 0.01 and ^∗∗∗^*P* < 0.001 versus original.

**FIGURE 6:**
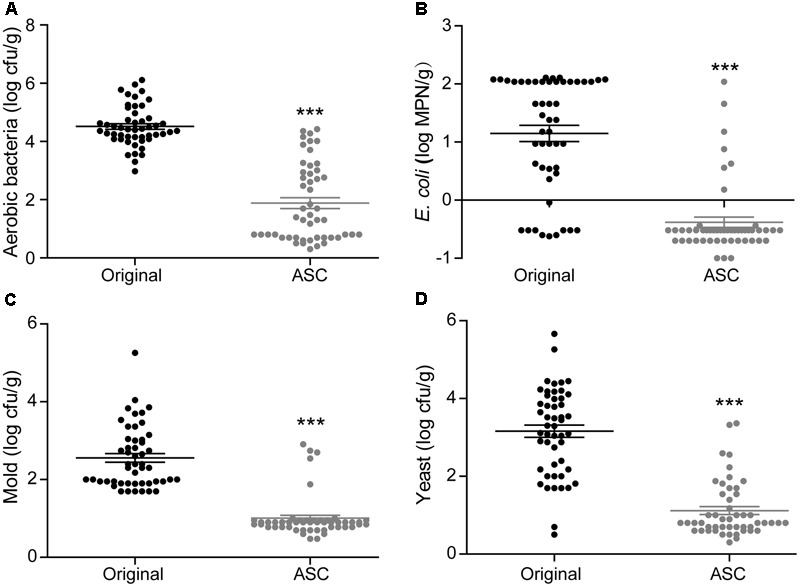
Effects of 0.1% ASC treatment on aerobic bacteria **(A)**, *E. coli* **(B)**, mold **(C)**, and yeast **(D)** for 50 randomly collected cherry tomato samples. Data are expressed as mean ± SD (*n* = 50). ^∗∗∗^*P* < 0.001 versus original.

**FIGURE 7:**
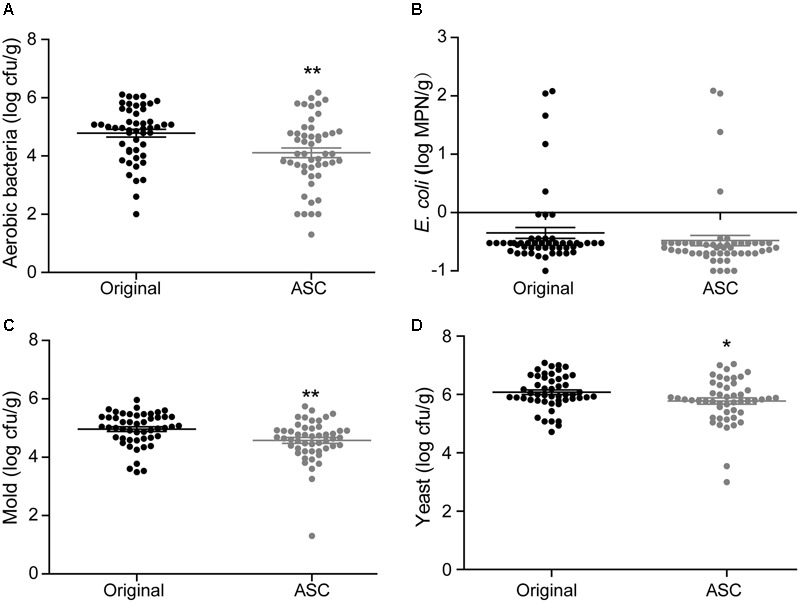
Effects of 0.1% ASC treatment on aerobic bacteria **(A)**, *E. coli* **(B)**, mold **(C)**, and yeast **(D)** for 50 randomly collected red bayberry samples. Data are expressed as mean ± SD (*n* = 50). ^∗^*P* < 0.05 and ^∗∗^*P* < 0.01 versus original.

A total of 50 samples of each fruit type from different retail grocers were subjected to treatment with 0.1% ASC, and the natural microbiota were evaluated before and after treatment. With the exception of *E. coli* on red bayberry, significant reductions were obtained for all microbiota and pathogens. The average microbial reductions on strawberry were 2.14 log cfu/g for aerobic bacteria, 0.40 log cfu/g for *E. coli*, 1.33 log cfu/g for mold, and 1.27 log cfu/g for yeast (**Figure 5**). The average microbial reductions on cherry tomato were 2.63 log cfu/g for aerobic bacteria, 1.53 log cfu/g for *E. coli*, 1.55 log cfu/g for mold, and 2.04 log cfu/g for yeast (**Figure 6**). The average microbial reductions on red bayberry were 0.68 log cfu/g for aerobic bacteria, 0.13 log cfu/g for *E. coli*, 0.39 log cfu/g for mold, and 0.30 log cfu/g for yeast (**Figure 7**). The reductions in natural microbiota were much higher for cherry tomato and strawberry than for red bayberry. The ASC application results on the three produce types from retail grocers were consistent with the sanitizing methods comparisons on different fresh fruits in this study. The sanitization of red bayberry with ASC solution is made difficult by the rough surface of red bayberry fruit. Although the reductions in *E. coli* obtained on the studied fruits were less than 1.55 log cfu/g, this does not indicate low sanitizing efficiency. *E. coli* contamination on the fresh produce samples was efficiently inactivated by ASC treatment, similar to the results obtained in the screening of sanitizing methods. The low reductions in *E. coli* on the retail grocers samples can be attributed to low contamination levels. *S.* Typhimurium contamination was not observed on any of the samples.

As indicated by López-Velasco et al. (2012) and other studies, fresh produce (including tomato) is usually washed by chlorinated water [e.g., 50–200 mg L^-1^ of active chlorine or active ClO_2_ (>3 mg/L)] to inactivate *Salmonella* spp. and other microbial contaminants. However, chlorine residue is limited in foods because of its toxic by-products (Richardson et al., 1998; Gil et al., 2016). In this study, chlorinated antimicrobial agents (i.e., NaClO and SC) were selected as the conventional sanitizing agents according to the EFSA Opinions on *Salmonella* and Norovirus [EFSA Panel on Biological Hazards (BIOHAZ), 2014]. GRAS organic acids (i.e., CA, LA, and MA) and ASC were selected as new methods for comparison. In addition to *Salmonella* spp., the effects of the traditional and new treatment methods were evaluated for pathogenic *E. coli* O157:H7, yeast, mold, and aerobic bacteria on strawberry, cherry tomato, and red bayberry. The results indicate that treatment with 0.1% ASC had the best sterilizing effect on these fruits; ASC treatment also required the shortest sanitizing time and low chlorite content. This method was further confirmed by examining 50 samples of each produce type.

## Conclusion

In this study, sanitizing with ASC strongly reduced natural microbiota and pathogens on strawberry and cherry tomato and produced moderate microbial reductions on red bayberry. A comparison of the tested sanitizing methods suggested that the sanitizing efficiency decreased in the order of 0.1% ASC (2 min) > 2% LA (10 min) > 0.02% SH (2 min) > 0.1% SC (10 min). Furthermore, compared to the other methods, treatment with ASC required a shorter sanitizing time and a lower chlorite content, which are important for maintaining the fruit texture and ensuring food safety. After treatment with ASC, treatment with 2% LA is the next best candidate for sterilizing the three fruits examined in this study because of its relatively high sterilization activity and GRAS certification.

## Author Contributions

YZ conceived and designed the experiments; WWe, XW, WWa, and YL performed the experiments; WWe drafted the manuscript; WWe, YZ, JX, and HG analyzed the data; YZ and ZX revised the manuscript. All authors read and approved the final manuscript.

## Conflict of Interest Statement

The authors declare that the research was conducted in the absence of any commercial or financial relationships that could be construed as a potential conflict of interest. The reviewer MS and handling Editor declared their shared affiliation.

## Abbreviations

ASC: acidified sodium chlorite
CA: citric acid
ClO^2-^: chlorine dioxide
FDA: Food and Drug Administration
GRAS: generally recognized as safe
LA: lactic acid
MA: malic acid
SC: sodium chlorite
SH: sodium hypochlorite
TW: tap water
